# Spatio-temporal changes of AOD in Xinjiang of China from 2000 to 2019: Which factor is more influential, natural factor or human factor?

**DOI:** 10.1371/journal.pone.0253942

**Published:** 2021-08-19

**Authors:** Jinglong Li, Qing He, Xiangyu Ge, Alim Abbas, Lili Jin

**Affiliations:** 1 College of Resources and Environment Sciences, Xinjiang University, Urumqi, Xinjiang, China; 2 Institute of Desert Meteorology, China Meteorological Administration, Urumqi, Xinjiang, China; 3 Key Laboratory of Oasis Ecology, Xinjiang University, Urumqi, Xinjiang, China; 4 Key Laboratory of Smart City and Environment Modelling of Higher Education Institute, College of Resources and Environment Sciences, Xinjiang University, Urumqi, Xinjiang, China; 5 Department of Atmospheric Sciences, Yunnan University, Kunming, China; Italian National Research Council (CNR), ITALY

## Abstract

Aerosol optical depth (AOD), which represents the optical attenuation, poses a major threat to the production activity, air quality, human health and regional sustainable development of arid and semi-arid areas. To some degree, AOD shows areal air pollution level and possesses obvious spatio-temporal characteristics. However, long-time sequences and detailed AOD information can not be provided due to currently limited monitoring technology. In this paper, a daily AOD product, MODIS-based Multi-angle Implementation of Atmospheric Correction (MAIAC), is deployed to analyze the spatio-temporal characteristics in Xinjiang Uygur Autonomous Region from 2000 to 2019. In addition, the importance of influencing factors for AOD is calculated through Random Forest (RF) Model and the propagation trajectories of pollutants are simulated through Hybrid Single-Particle Lagrangian Integrated Trajectory (HYSPLIT) Model. Spatio distribution of AOD presents a tendency that AOD value in northern Xinjiang is low while the value in southern Xinjiang is high. Regions with high AOD values are mainly concentrated in Tarim Basin. AOD in southern Xinjiang is the highest, followed by that in eastern Xinjiang and AOD value in northern Xinjiang is the lowest. Seasonal variation of AOD is significant: Spring (0.309) > summer (0.200) > autumn (0.161) > winter (0.158). Average AOD value in Xinjiang is 0.196. AOD appears wavy from 2000 to 2014 with its low inflection point (0.157) appearing in 2005, and then increases, reaching its peak in 2014 (0.223). The obvious downward tendency after 2014 shows that the use of coal to natural gas (NG) conversion project improves the conditions of local environment. According to RF Model, NG contributes most to AOD. HYSPLIT Model reveals that aerosol in southern Xinjiang is related to the short-distant carriage of dust aerosol from the Taklimakan Desert. Aerosol there can affect Inner Mongolia through long-distant transport. Blocked by the Tianshan Mountains, fine dust particles can not cross the Tianshan Mountains to become a factor contributing to AOD in northern Xinjiang.

## Introduction

As a primary element of the atmosphere, atmospheric aerosol is the generic term of heterogeneous system made up of solid and liquid particles suspending in the gas [1], whose mass is only a billionth of that of the atmosphere. Aerosol will not only influence the global climate directly [2] and indirectly [3, 4] by taking in and diffusing sun radiation, but is likely to cause harm to human health by spreading hazardous substance [2, 4, 5]. AOD is one of the fundamental optical parameters, which can be taken as an indicator of the level of local air contamination in some degree. Aerosol is used extensively as an uncertain but important indicator of the research on climate change and radiation equilibrium of atmosphere [6–8]. Therefore, AOD exerts great influence on climate regionally and even globally, atmospheric radiation transmission, as well as water circulation [9–11]. In the troposphere, dust aerosols account for one-third of atmospheric aerosols [12] and the strong heating or cooling effect caused by its changes will alter the thermal condition of the atmosphere, therefore affecting the dynamic structure of the atmosphere [13].

In the conventional sense, a main way of AOD acquisition is field exploring, which neither shows continuity in space nor satisfies the demands of regional study because of restrictions on the allocation of observation posts faced by ground survey. At present, remote sensing (RS) technology is a crucial mode to test and supervise aerosol on the strength of AOD [13–22]. As a cylinder set completion, AOD reflects the load of aerosol column and aerosol’s effect on the earth radiation budget (An). Owing to its distinct advantages, RS offers an executable way to acquire AOD with high time resolution on a large special scale, which overcomes problems such as the absence of ground observation data and spatio inhomogeneity, providing references to fully understand the distribution and concentration of aerosol and theoretically supporting regional atmospheric environment control. AOD information searched by using MODIS has the advantage of global distribution, whose coverage seems to be a daily or continuous occurrence, estimating aerosol’s characteristics in Xinjiang region, and finding the limitation of estimated algorithm [23]. MODIS products were applied to analyze seasonal dynamics of photometric characteristics of aerosols in East Asia from 2000 to 2005 [24], while most studies on AOD were on a rough spatio resolution scale and very few studies were based on arid and semi-arid regions. Besides, there was a concern over the accuracy of inversion products. No observation data was used to evaluate these products especially in areas lack of data (such as Xinjiang). Therefore, it is essential to conduct product verification and product-based spatio-temporal analysis. MCD19A2 is used to study AOD with high resolution in Xinjiang, because MCD19A2 data integrates TERRA and ACQUA together and adopts MAIAC algorithm, which has the characteristics of high resolution, wide range and high accuracy of inversion (reaching 1km). Obtaining AOD data in Xinjiang can not only make up for the shortage of long time series of AOD data in the north-western of China, but also provide data persistence for the studies on the spatio-temporal changes of AOD in Xinjiang in the future.

Xinjiang Uygur Autonomous Region (briefly named Xinjiang) in northwest China is a portion of the sandstorm zone in Central Asia, which is among the major origins of dust aerosols [25]. Xinjiang is situated at the hinterland of the Eurasia. With the economic development of northwest China, severe air pollution caused by human activities and industrial development makes atmospheric environment in Xinjiang become worse and worse. Since 2013, the coal-to-NG conversion project in Xinjiang has helped curb pollution, but the situation still remains grim. The Cloud-Aerosol Lidar with Orthogonal Polarization (CALIOP) inversion shows that the altitude of the dust aerosol layer in the Taklimakan Desert is 4–5 km, and the vertical distribution of color ratio and particle depolarization indicates that dust is important [26]. In 2016, the air pollution in southern Xinjiang presented obvious characteristics of seasonal variability and spatio pattern. Besides, the concentrations of PM_10_ and PM_2.5_ sharply increased because of sandstorms [27]. PM_10_ was the major pollutant influencing quality of the western air in China [25]. Atmospheric dust-fall study in Urumqi showed total suspended particulate (TSP) was the major pollutant, which was brought to this region by north-west wind in spring and summer [28]. Various aerosols exist in Duschanbe, Tajikistan, obviously affecting climate in Central Asian, Tianshan Mountains, Tibet Plateau and the rest of the world [29]. The increase of aerosol load in Central Asia is caused by the influence of precipitation and dust on aerosols from arid regions. The biggest contribution of mixed aerosols is created in spring and summer [30]. These results show that contribution of dust to aerosol in Xinjiang and Central Asia is significant, and the spread and migration of aerosol affect the whole Xinjiang and Central Asian, which is of practical significance. However, research on spatio-temporal distribution and driving force analysis of long-time AOD in southern Xinjiang, northern Xinjiang and eastern Xinjiang is limited. Therefore, studies on spatio-temporal variation of aerosol in Xinjiang, AOD trajectory simulation and driving force analysis in areas with high aerosol contribution will have significant influence on climate change and ecological environment in adjacent regions of northwestern China and central Asia.

This paper targets for (1) studying spatio-temporal characteristics of aerosol in Xinjiang (southern Xinjiang, northern Xinjiang and eastern Xinjiang) from 2000 to 2019, (2) ranking the importance of AOD influencing factors combined with multiple influencing factors, (3) stimulating AOD trajectory distribution in areas with high AOD values, and (4) exploring correlated features of aerosol in Xinjiang.

## Study area and data collection

### Study area

The natural area of Xinjiang (73°40′E~96°23′E, 34°22′N~49°10′N) consists of southern Xinjiang, northern Xinjiang, and eastern Xinjiang (https://eol.jsc.nasa.gov/SearchPhotos/). Northern Xinjiang is from the south of Northern Altai Mountains to Tianshan Mountains including Junggar Basin, Lli Valley and other areas. Southern Xinjiang includes the south of Tianshan Mountain and the north of Kunlun Mountain, mainly dominated by Tarim Basin, Tarim River and Taklimakan Desert. Eastern Xinjiang is mainly to the east of Hami city, Balikun, Tokeson and other six counties and cities, and adjacent to Jiuquan City, Gansu Province. Located in the North Temperate Zone, Xinjiang possesses “temperate continental climate”, which is mainly characterized by “cold in winter and hot in summer, wide daily and annual temperature range, and rare annual precipitation”. Its latitude across the north and south is 15°. Due to the significant difference of terrain elevation, southern Xinjiang is warmer than northern Xinjiang, and western Xinjiang is drier than eastern Xinjiang. Being far from the sea and affected by its terrain, Xinjiang rarely receives moisture from the Pacific and Indian oceans, whose precipitation mainly results from moisture from the Atlantic Ocean brought by west wind, and a bit of moisture from the Arctic Ocean.

### MCD19A2-MODIS AOD data

There are 36 discrete spectrum bands in the MODIS sensor, from 0.41 to 14.5μm, covering a geographical range of 1200 km×1200 km, and its spatio resolution is 1 km. Combining MODIS Terra and Aqua, MCD19A2 (https://ladsweb.modaps.eosdis.nasa.gov/) is a kind of MAIAC terrestrial AOD secondary grid product, which is able to extract AOD data of ocean and land efficiently and accurately, and is applicable for bright undersurfaces such as desert, plateau and arid areas [31]. MODIS aerosol data products loaded by using the AOD day-to-day product information of MCD19A2 were projected for conversion and tessellation through the MRT tool. Daily mean value was used to synthesize AOD data monthly to ensure that the surface reflectance was more realistic. Mean value of effective pixels was calculated taking pixel as its processing unit (ignoring the filling value and error value). First, vector data was applied to cut out the regional unit of Xinjiang, and then corresponding annual value, monthly value and quarterly value were obtained. Finally, the plot with ArcGIS 10.3 was performed (ESRI, Redlands, California, USA).

### Statistical yearbook data

According to previous studies, aerosol was mainly limited by weather conditions, transportation, energy structure and other human-related causes [32–34]. Therefore, given the natural and human influence, 10 main parameters were chosen consisting of temperature(T), precipitation(P), sunlight hour (SH), heating coal consumption (HC), motor vehicle ownership (MVO), number of private cars (NPC), natural gas usage (NG), total industrial consumption (TIC), population (PL), and urban green space (UGS) as key parts affecting atmospheric aerosol concentration. All the data applied in this paper was from China City Statistical Yearbook (http://www.stats.gov.cn/tjsj/ndsj/) and Xinjiang Statistical Yearbook.

### Ground-based AOD data

**Fig 1:**
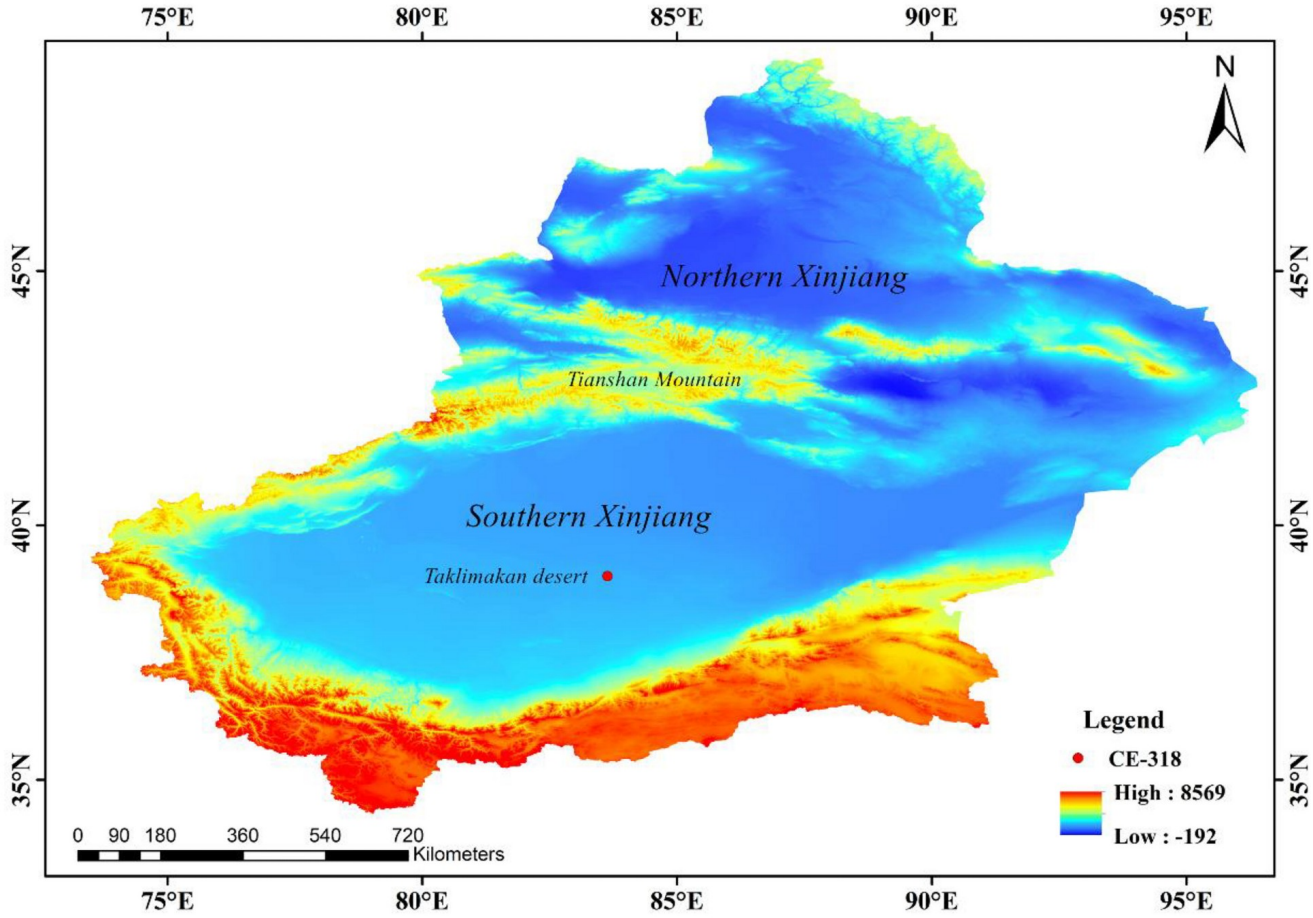
Location of study area (The red is the highest value and dark blue represents the lowest value. Map is created using ArcGIS version 10.3. Xinjiang Uygur Autonomous Region is downloaded from The Gateway to Astronaut Photography of Earth website (https://eol.jsc.nasa.gov/SearchPhotos/) Map credit: Jinglong Li).

A CE-318 sun sky lunar multispectral photometer is situated at Atmospheric Environment Observation and Experiment Station of Taklimakan Desert, which belongs to China Meteorological Administration and is 200 kilometers away from the deep area of Taklimakan Desert (38°58′N, 83°39′E, 1090 m above the sea level), as is shown in Fig 1.

This study was carried on AOD observations by using 8-waveband CE-318 sun sky lunar multispectral photometer (Cimel Electronique, France). And this instrument can measure solar and sky radiation with higher precision, consisting of aerosol bands with 440nm, 670nm, 870nm, and 1020nm, water-vapour band with 936-nm, and three polarization bands with 870-nm. Each channel has a bandwidth of 10 nm. AOD data on the ground corresponded to the average figure of 30 min around the satellite transmission time. This information was applied to calculate the AOD value of 550nm band through Angstrom Exponent, and was in comparison with the AOD value of MODIS products, so as to contain validation information of MODIS products. The calculation of AOD values is as below:

$$\tau =\;\beta \varepsilon^{-\alpha },$$

AOD is the τ (ε) and Angstrom exponent is the α that is the ratio of big or small particles in the aerosol ingredient, and Angstrom turbidity coefficient is the β which is used to make the aerosol concentration measurement in the atmosphere.

The Angstrom exponent computing calculation is as below:

$$\alpha =\;\frac{ln[\tau (\lambda_{1})/\tau (\lambda_{2})]}{ln(\lambda_{1}/\lambda_{2})}$$

AOD is the *τ* (*λ*). The wavelengths is and *λ*_1_ and *λ*_2_ (nm).

### Random Forest model

Random Forest (RF) model is a tool for studying algorithm, which is applied in category and regression using the concept of holistic learning to integrate multiple decision trees. The RF algorithm can cope with high-dimensional data and can be applied in gather. This model includes many numbers of trees [35]. RF selection is used to choose partition variables of the minimized set for regression, whose output is the mean value of all decision-making trees [36, 37]. RF uses multi-variate and several specimens, sorts the determinant of variables, and provides relative significance [38]. Besides, as for out-off- balanced samples, RF can balance the error and prevent it from overfitting and decreasing generalization error. AOD is a dependent variable. 10 concomitant variables consisting of temperature(T), precipitation(P), sunlight hour (SH), heating coal consumption (HC), motor vehicle ownership (MVO), number of private cars (NPC), natural gas usage (NG), total industrial consumption (TIC), population (PL), and urban green space (UGS) are independent variables (T, P, SH are natural factors, and the others are human factors). Within the framework of RF model, concomitant variables are used to evaluate AOD value, and the significance of concomitant variables is obtained.

### The Hybrid Single-Particle Lagrangian Integrated Trajectory (HYSPLIT) model

Hybrid single-particle Lagrangian integrated trajectory (HYSPLIT) is a useful instrument to set up the domain of space of air parcels to the receptor places [39–43]. This model possesses complete modes of transport, diffusion and settlement to deal with various input field of meteorological elements, diversified physical processes and different pollutant discharge sources, which is widely used in the analysis of air pollutant transportation [41, 44, 45]. Based on HYSPLIT model, the forward and backward trajectory simulation of the high-value AOD region in Xinjiang was carried out to get the source and spread of AOD in this region. The software and data were downloaded from the NOAA website (http://www.ready.noaa.gov/documents/Tutorial/html/install_win.html and http://arlftp.arlhq.noaa.gov/pub/archives/gdas1/).

## Results and analysis

### MODIS product verification

**Fig 2:**
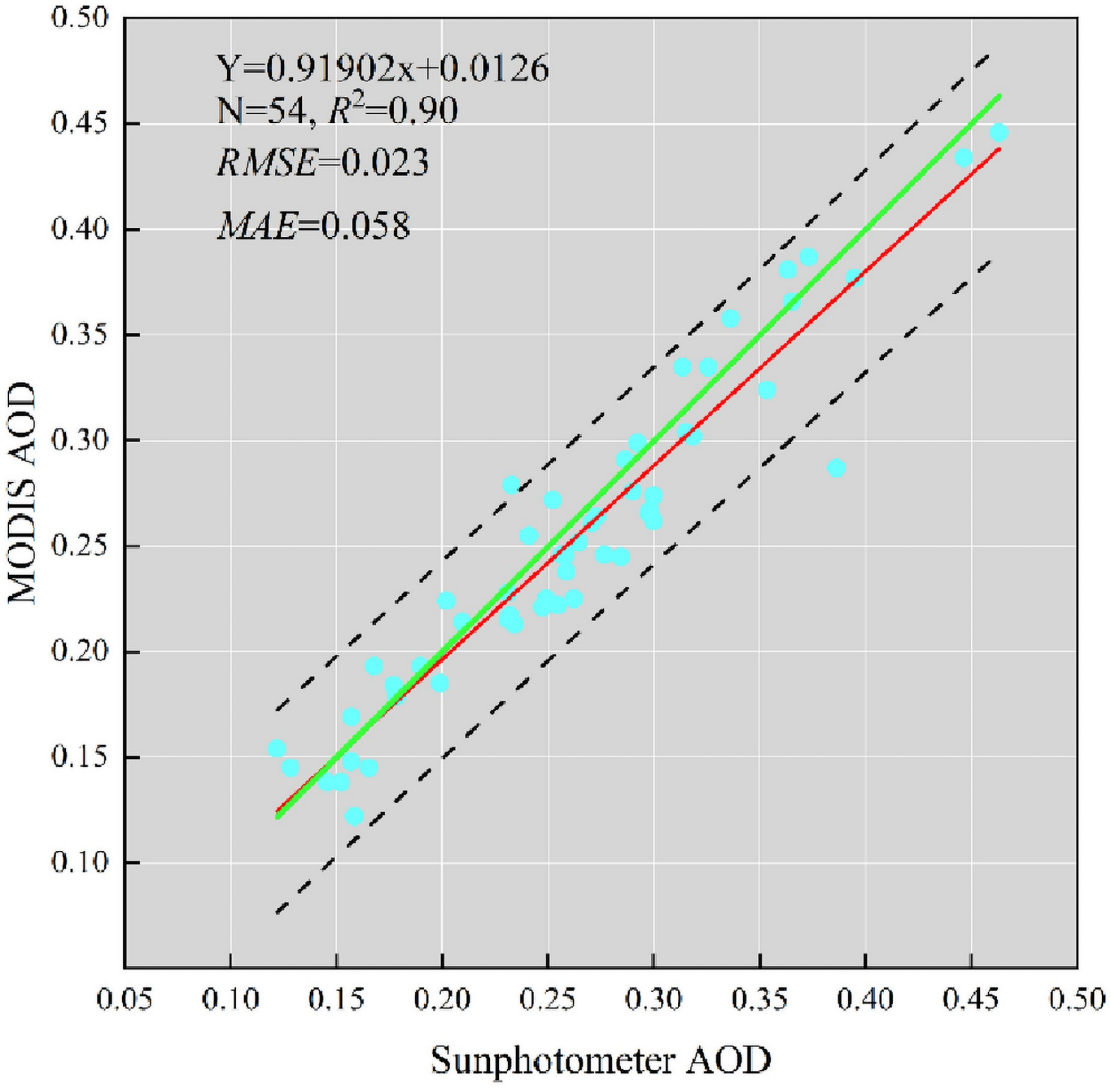
MODIS AOD product datum is compared with filed observations by solar photometers. The green line is the 1:1 line, the red line is the regression line and the black dashed lines indicate the 95% confidence range.

The results of MODIS aerosol product (MCD19A2) were matched with the measured data of CE-318 solar photometer for accuracy verification. Generally speaking, ground-based observing has the quality of high-precision and is extensively applied to AOD verification [46, 47]. Decisive coefficient (R^2^), root mean square error (RMSE) and mean absolute error (MAE) indicators are applied to value the implementation of the estimates. As is shown in Fig 2, the continuity of ground AOD data and the MODIS AOD data are better than ever, which have high coefficient of association (R = 0.953), conclusive coefficient (R^2^ = 0.90) and low root-mean-square error (RMSE = 0.023) and MAE (0.058). Fitted curve and 1:1 curve have good coincidence, especially in the low value range. So far, MODIS AOD products have been caught on and used by a lot of scholars [48–50].

### Characteristics of the spatio distribution of AOD

**Fig 3:**
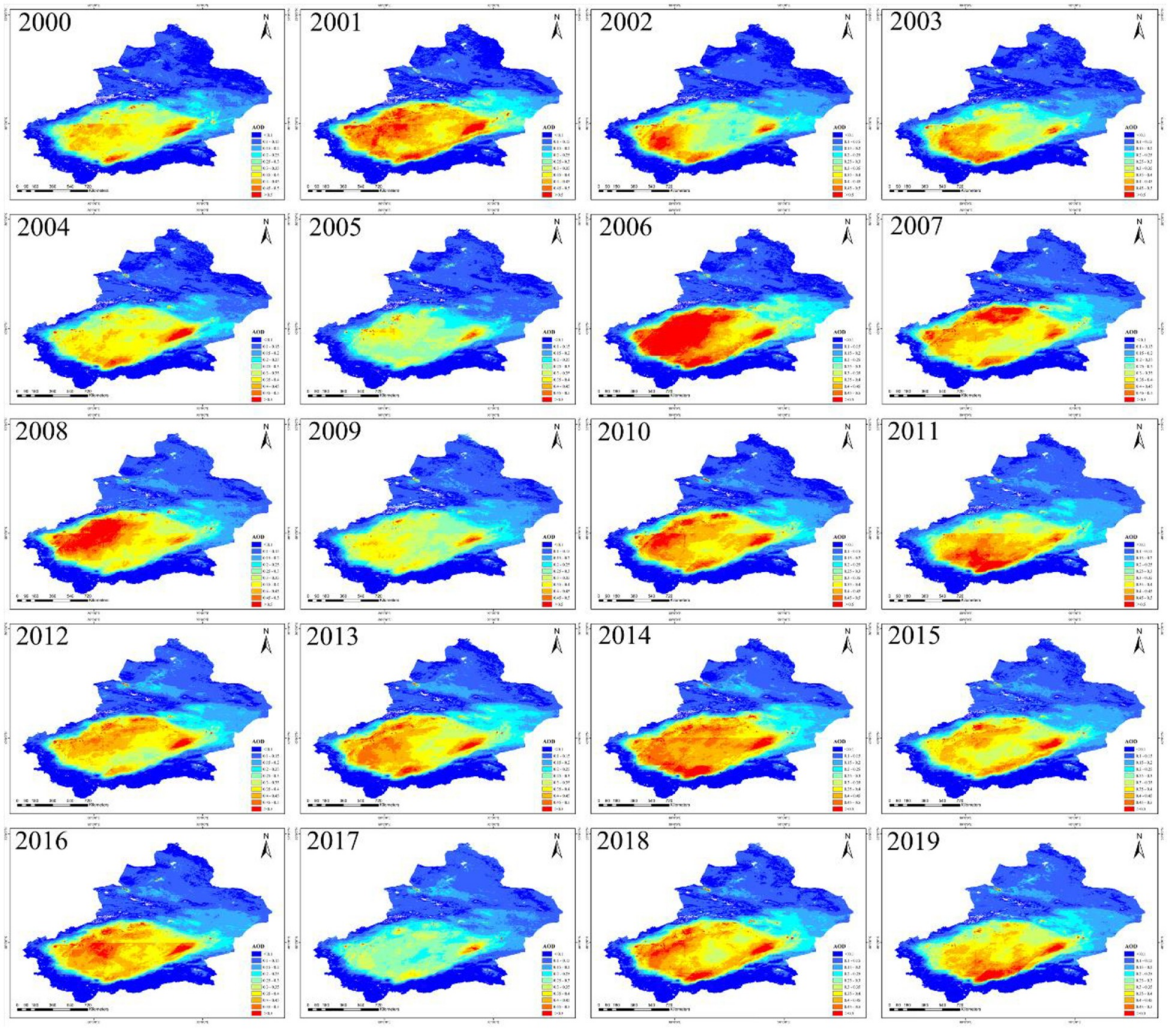
Spatio distributions of AOD of 2000–2019 (The red is the highest value and dark blue represents the lowest value. Map is created using ArcGIS version 10.3. Xinjiang Uygur Autonomous Region is downloaded from The Gateway to Astronaut Photography of Earth website (https://eol.jsc.nasa.gov/SearchPhotos/) Map credit: Jinglong Li).

Spatio distribution of AOD in different years in Xinjiang is shown in Fig 3. AOD in eastern Xinjiang and northern Xinjiang kept the value uniformly low from 2000 to 2019. AOD in southern Xinjiang kept the value high from 2000 to 2019 due to the high contribution of dust particles in Taklimakan Desert to AOD. Fig 5a shows the relevant statistical information of the spatio distribution of AOD in Xinjiang. AOD appeared wavy from 2000 to 2019, during which, there was a slight rise and a gradual decline from 2000 to 2005 (0.171–0.206–0.158); low inflection point (0.158) appeared in 2005, and then AOD obviously increased. Maybe this is related to the promotion and deployment of the economic development strategy as well as the increase of coal-fired heating (burning fossil fuels) throughout Xinjiang. After 2014, AOD revealed a significant fall, which may result from the strengthened environmental awareness of local people and the macro-control policies of “energy saving and emission reduction”, “preservation of environment” and “low-carbon trip” proposed by local government. Xinjiang includes southern Xinjiang, northern Xinjiang and eastern Xinjiang according to its natural regions. AOD in southern Xinjiang presents high AOD distribution possibly because it is located beside the Taklimakan Desert in Tarim Basin. AOD around the Desert has a tendency to increase as it is a concentrated area for sand aerosol. High contribution of sand aerosol is caused by the local dry weather with sparse rainfall, especially annually 157-day dust weather accompanied by dust storms and wind whirl. AOD value in northern Xinjiang and eastern Xinjiang remained low and were lower than the average AOD value from 2000 to 2019, which possibly has a connection with less sand disturbances and higher regional vegetation coverage.

**Fig 4:**
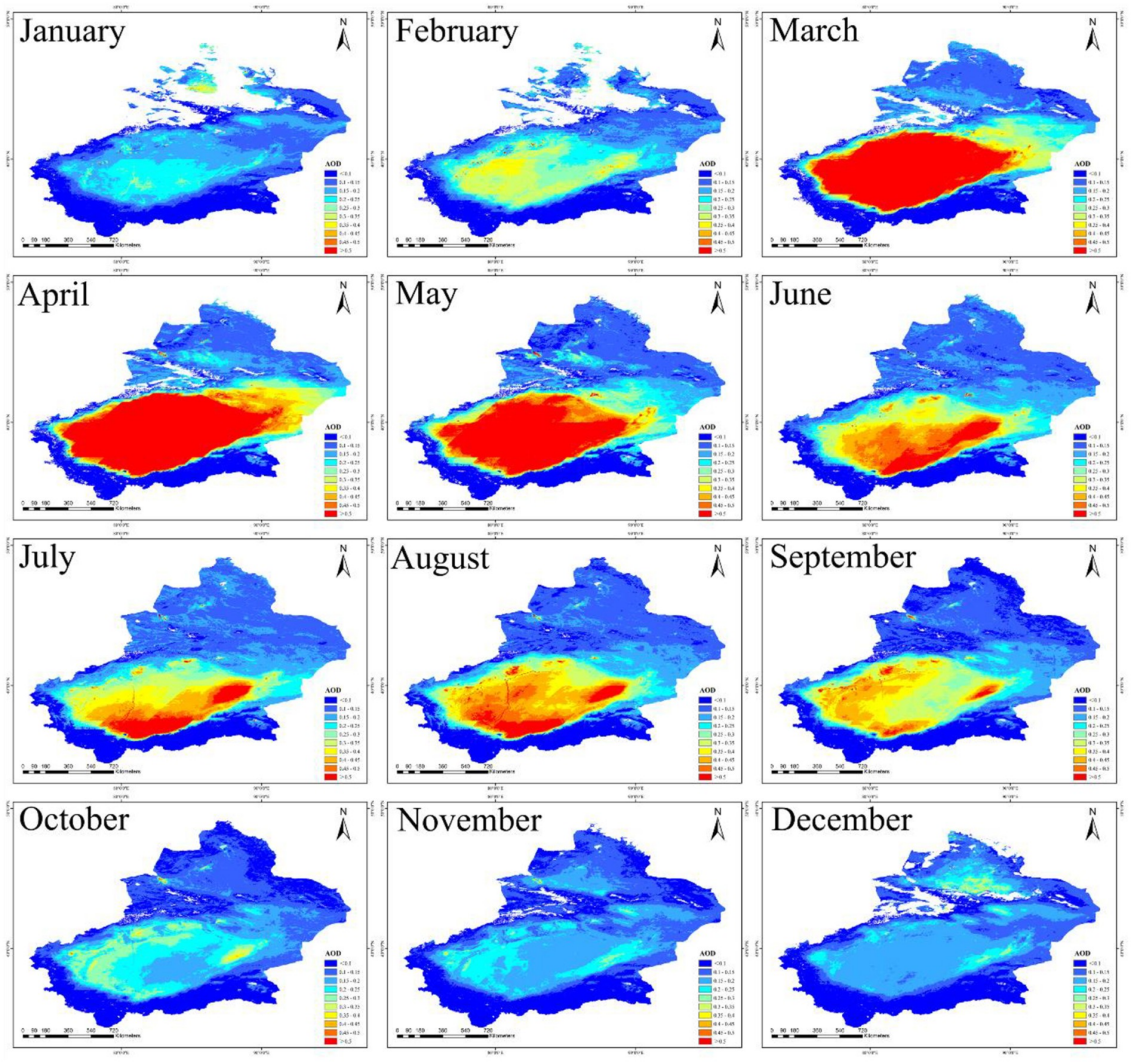
Spatio distribution of average AOD in different months (The red is the highest value and dark blue represents the lowest value. Map is created using ArcGIS version 10.3. Xinjiang Uygur Autonomous Region is downloaded from The Gateway to Astronaut Photography of Earth website (https://eol.jsc.nasa.gov/SearchPhotos/). Map credit: Jinglong Li).

**Fig 5:**
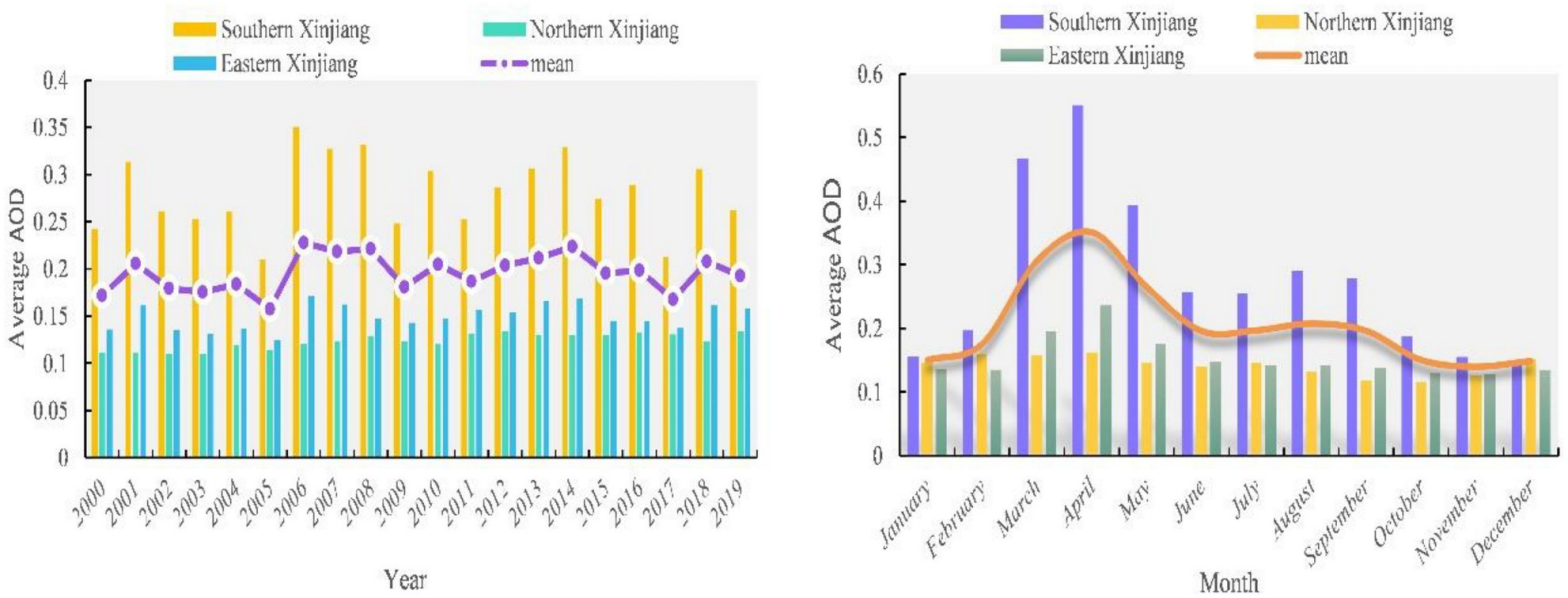
(a)The dynamic alteration of AOD in the research of region from 2000 to 2019, and (b) spatio spread of the mean AOD in different months from 2000 to 2019.

Fig 4 shows the monthly spatio distribution of AOD. High AOD value in Xinjiang appeared in April (0.351) and its low value appeared in December (0.148). Fig 5b shows that the monthly change of AOD in Xinjiang appeared an obvious “unimodal” curve. The 12-month change rule of AOD value in southern and eastern Xinjiang appeared “sharp rise—significant decline—slow decline” (0.156–0.551–0.147; 0.136–0.236–0.135), generally consistent with the changing trend in Xinjiang (0.150–0.351–0.149). Both reached their peaks in April because of large numbers of windy and dusty days. Monthly AOD in northern Xinjiang changed little, due to the fact that Xinjiang Development Center is located in northern Xinjiang where policies are better implemented and regional environment are better protected. One interesting phenomenon found was that Tianshan Mountains blocked the regions of northern Xinjiang and southern Xinjiang so that dust from southern Xinjiang could not cross Tianshan Mountains to affect AOD value in northern Xinjiang, and only spread around Tarim Basin. As a result, the aerosol in Xinjiang was lower in the north and higher in the south.

### Seasonal time series of AOD

**Fig 6:**
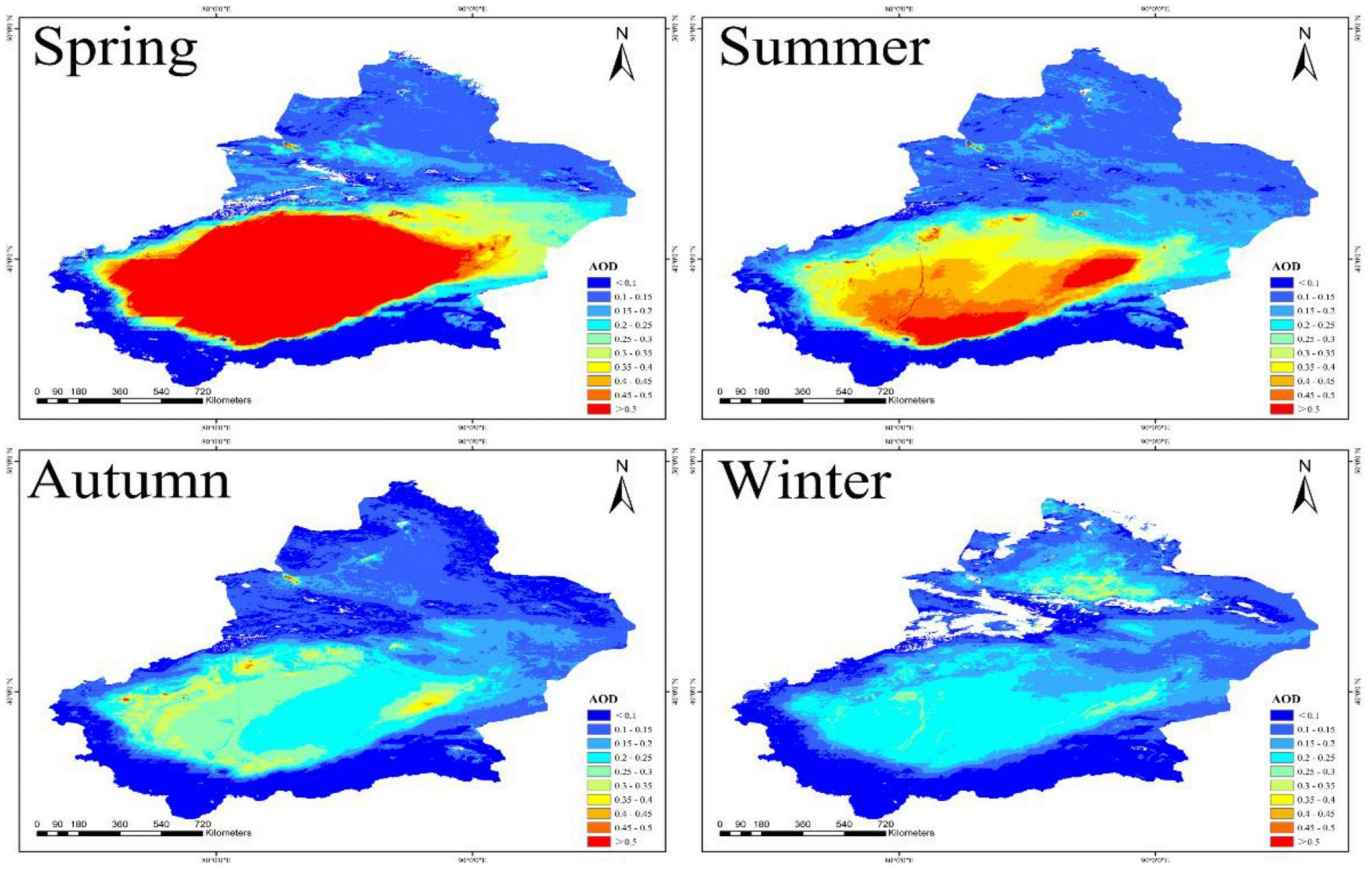
Seasonal mean AOD distribution (The red is the highest value and dark blue represents the lowest value. Map is created using ArcGIS version 10.3. Xinjiang Uygur Autonomous Region is downloaded from The Gateway to Astronaut Photography of Earth website (https://eol.jsc.nasa.gov/SearchPhotos/). Map credit: Jinglong Li).

**Fig 7:**
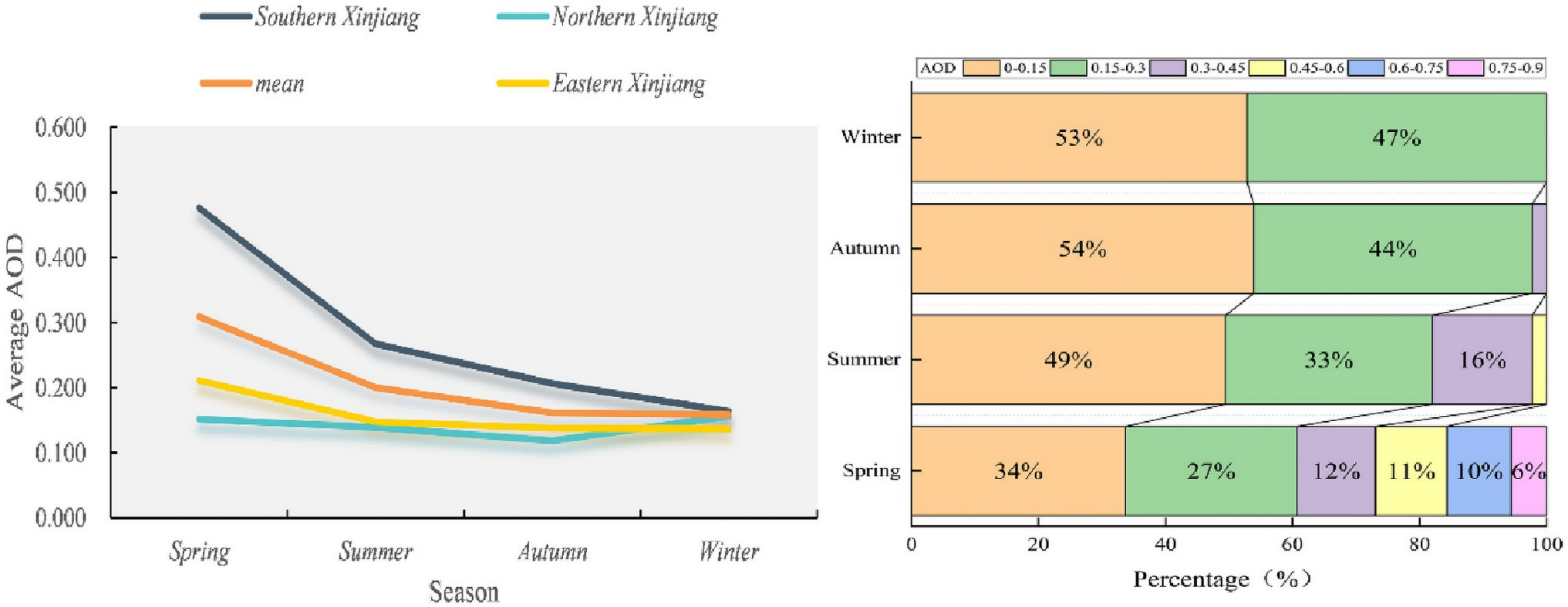
(a) Spatio distribution of the average AOD in each season (spring, summer, autumn, and winter) from 2000 to 2019, and (b) varying degrees of seasonal AOD variations.

The year is divided into four seasons based on the temperature change. March, April and May belong to spring, June, July and August make up summer, and so on [51]. Fig 6 shows the average seasonal spatio distribution of AOD from 2000 to 2019. High AOD value areas in spring were in southern Xinjiang (0.476), accounting for 42.8 percent of four seasons, and the AOD value in summer was 0.267, autumn 0.207 and winter 0.163, which decreased successively. AOD in northern and eastern Xinjiang remained low. High AOD value in spring was related to the dust in this season. In addition, as southern Xinjiang region was affected by the Taklimakan Desert, there were more windy days in spring. Wind speed accelerated the movement of mobile dunes so that dust particles in the atmosphere spread faster. Fig 7 shows the relative statistics information of seasonal AOD distribution. AOD value reached its maximum in spring (0.309), accounting for 37.2 percent. High temperature, good transparency and increased surface vegetation coverage in summer results in a decline in the number of aerosols getting into the atmosphere from the surface. The decrease of AOD in autumn was different from that in summer. Controlled by subtropical high for a long time, it was with stable conditions and good diffusion conditions. AOD showed a tendency of steep decline from spring to winter. The downward tendency in southern Xinjiang and eastern Xinjiang was consistent with that in the whole Xinjiang region, but the most obvious decline appeared in southern Xinjiang (0.476–0.163), which was also related to the sand and dust weather in spring in Tarim Basin. East of southern Xinjiang and eastern Xinjiang were high AOD value regions with strong winds, resulting in the eastern Xinjiang subject to dust transport and pollutant emission in spring, and it reached a high value (0.211), which was also consistent with the previous analysis results. Surprisingly, as we found, AOD in northern Xinjiang reached high value (0.156), accounting for 27.6 percent of the whole season. This was because the cold air blocked by the north side of Tianshan Mountains accumulated near Tianshan Mountain in this area, exacerbating the cold and causing central heating in the region to start before October 10 each year. Correspondingly, heavy coal burning for regional heating caused severe air pollution in winter.

### The importance of natural and perceived factors

**Fig 8:**
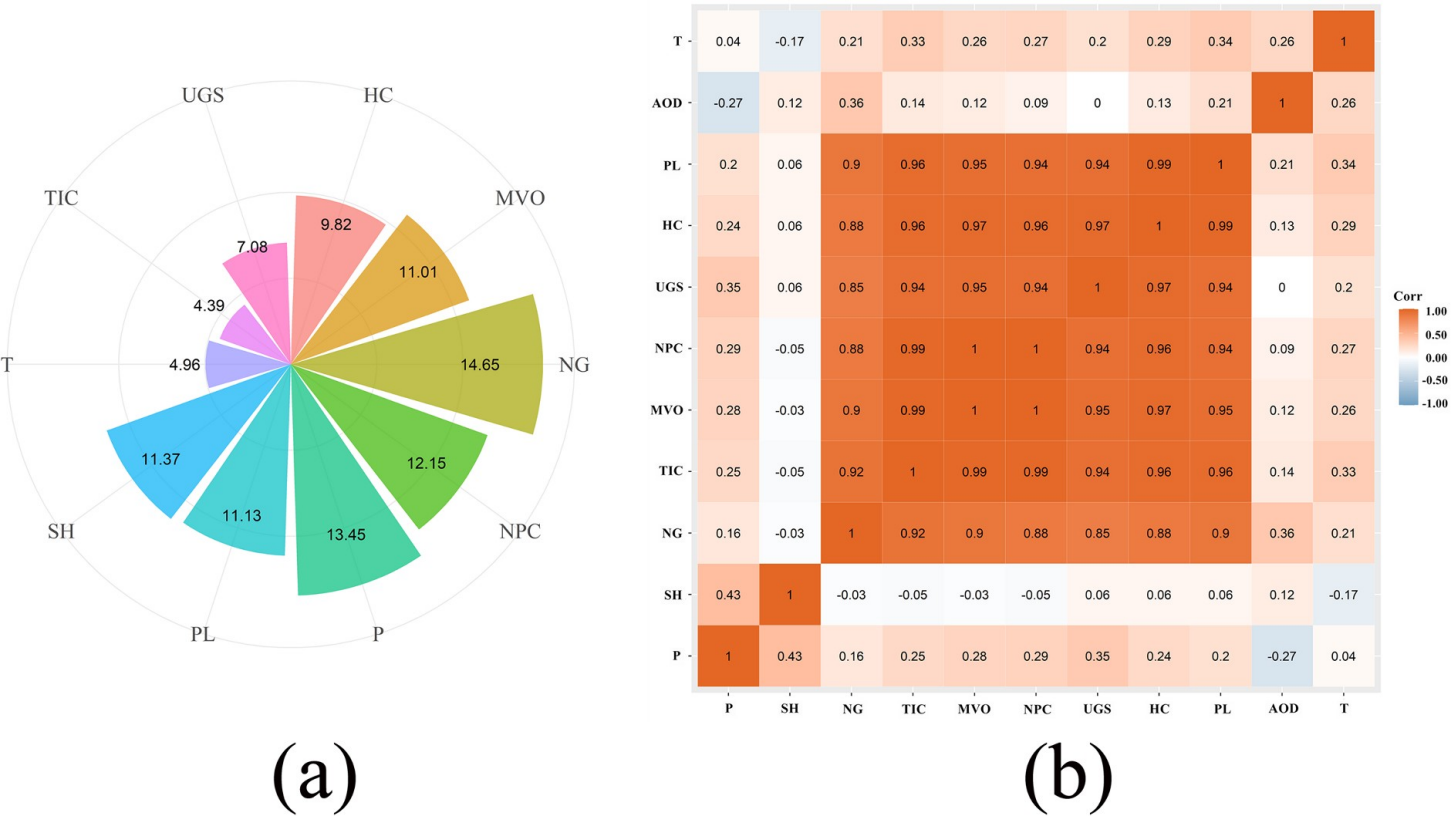
(a) Random Forest (RF) model simulation AOD renderings, (b) relevant coefficient between AOD and various variables in Xinjiang.

Major reasons affecting Xinjiang can be summarized as natural and human factors. 10 aggregative indicators were applied to study the principal consideration influencing AOD change. RF model was used to analyze the above 10 chosen indicators of quantitative assessment, and the result was shown in Fig 8a. The result showed that NG was the most significant human factor, accounting for 14.65 percent, and P was the most significant natural factor, accounting for 13.65 percent. In Fig 8b, the correlation coefficient of NG was as high as 0.36, with a significant positive correlation. The precipitation had an obvious negative correlation, reaching 0.27, which showed that NG and precipitation produced the greatest influence on AOD. Due to the cold weather in winter and early spring, the heating supply starts on October 10 and ends on April 15. Although the coal to NG conversion project reduces the local air pollution, NG burning discharges SO_2_, CO, NOX and other pollutants. Therefore, high contribution of AOD is also reasonable. Temperature and SH affected AOD by indirectly influencing the formation of clouds and decomposition of particulate matter. TIC directly reduced particulate matter entering the atmosphere to affect AOD, HC, MVO and NPC and other factors, thus having a direct or indirect effect on AOD. Population and UGS affected AOD by disturbing the local climate indirectly.

### Characteristics of the contaminant potential transport pathways

The study was on the grounds of meteorological data set from the National Center for Environmental Prediction (NCEP) and reanalysis data around the globe of the Global Data Assimilation System (GDAS), consisting of pressure, horizontal and vertical wind speeds, temperature, and relative humidity. MeteoInfo was used to stimulate the forward and backward trajectory of atmospheric pollutants, and potential source and transportation path of pollutants in spring of 2014 in southern Xinjiang was obtained. Major steps were as follows: first, the longitude and latitude for the starting position was determined. In this study the geographical position of the hinterland of the Taklimakan Desert (38.58 N, 83.39 E) in southern Xinjiang was the target location of the backward track and the beginning location of the forward track. Second, a beginning time limit of simulation was set. This paper simulated trajectory of the season with high AOD value (from March to May) in southern Xinjiang. Third, time and height for the stimulation were set up. The interval was 6 hours from the initial time, namely, the 48-hour forward and backward trajectory was simulated at 00:00, 06:00, 12:00, and 18:00 every day. 500 m was selected for the simulation height, as near-layer wind appeared at the height of 500m, and the mean flow field features of the boundary layer and then the average flow field features of the boundary layer and the mass transportation characteristics of gas in the near layer could be further reflected [52].

**Fig 9:**
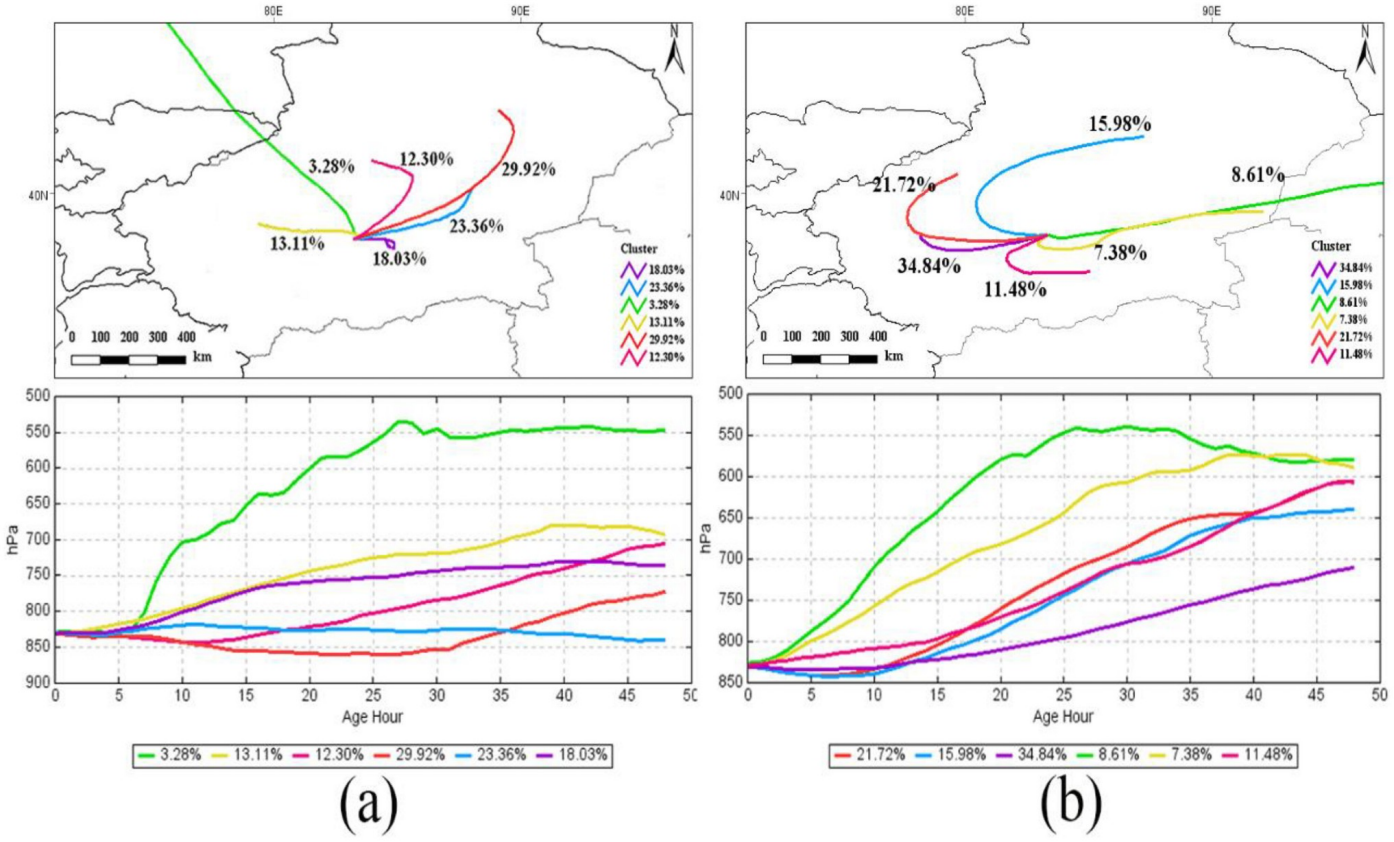
Analysis of the air parcel track from southern Xinjiang in the spring of 2014. (a) backward track and (b) forward track. (The lines in the figure represent paths and the colors represent percentages of clustering. The software is downloaded from http://www.ready.noaa.gov/documents/Tutorial/html/install_win.html.. Map credit: Jinglong Li).

Based on the airflow spatio similarity in the HYSPLIT model, cluster analysis method was used to analyze the potential transport path of pollutants in southern Xinjiang, and estimate potential transport direction and proportion of the trajectory. From Fig 9, it can be seen that fine dust particles contributed the most (accounting for 66.8%) when air quality was poor in spring in southern Xinjiang, which was mainly affected by internal dust sources. This is a significant aspect of the poor air quality in spring, as it is a season of high wind speed and frequent sandstorms. Pollutants in eastern Xinjiang accounted for 29.92%, and the dust sources outside were mostly Central Asian dust sources (3.28%), most of which were short distance transports. Grain diameter of particles reaching southern Xinjiang gradually decreased with the increase of the transportation distance.

The air parcel trajectories in southern Xinjiang were spread to the southwestern region, northwestern region and eastern region, accounting for 34.84%, 37.7% and 27.47% respectively. Pollutants moving to the southwest accounted for 34.84%, which was the shortest from its origin. These pollutants were mainly centered in a lower level above the origin surface, and the height of diffusion was about 1.5 km which settled in surrounding area. Within two days, the air parcels may influence eastern Xinjiang and some parts of Inner Mongolia. The wider the influenced ranges of the diffusion trajectory were, the lower the probability of occurrence was. The Tarim Basin was low in elevation (800–1300 m), and dust aerosols could only diffuse and transport in the interior due to the difference in height. Interestingly, 37.7 percent of diffusion trajectory moved from the west to the northwest in the medium term, but failed to enter northern Xinjiang due to the block of Tianshan Mountains with an average altitude of 5,000 meters.

## Discussion

Spatio resolution rate of MCD19A2-MODIS AOD data reached 1km, making up for the deficiency in dark target way and deep blue algorithm. For the surface of dark target, the dark target way has the quality of high precision. The accuracy of light color is better than that of dark color. But no matter whatever forms of aerosol, products need to be tested and verified, as there is an underestimation of remote sensing products, which can be commonly seen. Remote sensing data was only used to imitate or retrieve the actual surface and could not be in accordance with the actual value, which was also the primary source of errors, so the remote sensing goods needed to be verified. MOD08 products were applied to relative research on Taklimakan Desert [24]; however, by comparison with the observed values, there were also underestimates. Products in this study are more specific.

Monitoring of the ground in real time can fully reflect the changing tendency and spatio distribution of air quality within a certain range. Traditionally, analysis and study on atmospheric aerosol includes real-time ground monitoring. It is difficult for this method to meet the research needs because it can only reflect the variety and distribution features of AOD on a small scale near the monitoring site, and it is also hard to realize spatio real time continuity besides the high data acquisition cost. Remote sensing technology can make up for it and solve the problems of short of measured data and unbalance spatio distribution, and is characterized by greater efficiency, larger scale and less cost. It shows the distribution features, diffusion and propagation path of large-scale atmospheric pollutants, so that we may get large-scale AOD data with high temporal resolution [53, 54]. AOD is a day-to-day composite product data set, which is synthesized by using the daily mean value, thus well avoiding the influence of external environment and making the data more reliable.

AOD is significantly affected by different environmental factors. Analysis of AOD spatio-temporal showed that AOD level reached its maximum in spring. In fact, AOD level in spring was high and changeable, which mostly had to do with the uncertainty of windy weather and ground features. Spring is characterized by warmer land, melting snow, more exposed and loosen soil, as well as windy weather which allows dust to float on the exposed soil and particle to increase concentration in the air. Given the above reasons, we need to quantify AOD in such situation in future studies.

HYSPLIT model was applied to simulate the forward and backward track of the AOD high value region. It was found that major AOD contribution source area was in Taklimakan Desert in the Tarim Basin where there were frequent windy and sandy days throughout the year, especially in the spring. The local wind power would produce thermal differences when the local temperature increased. Uniform heating and temperature differences led to strengthening turbulent exchange. When wind speed increased to a certain threshold, more sands and dust would enter the air, resulting in an increase in aerosol concentration. Dust aerosol contributed little to northern Xinjiang as the dust particles in the upper air were blocked by the Tianshan Mountains with an average altitude of 5000 meters. The transportation of dust aerosols was restricted by height. Therefore, the major aerosol contribution sources in northern Xinjiang, southern Xinjiang and eastern Xinjiang need to be quantified respectively in the future.

At present, how to take valid and efficient measures to prevent further aggravation of air pollution has attracted the attention of the academia and the government. Serious air pollution will do harm to human health, and even restrict the regional sustainable development. This paper used RF model to conduct analysis of driving force of AOD in Xinjiang and found UG and precipitation produced a server influence on AOD. In recent years, China has given priority to the development of various industries in Xinjiang. As the central region of the Silk Road economic belt of “One Belt and One Road”, Xinjiang has witnessed the continuous growth of coal mining, coal power, coal chemical industry, petroleum and natural gas chemical industry as well as non-ferrous metal mining and processing, which have actually caused air pollution in Xinjiang. Air pollution can increase morbidity of disease of respiratory system, and even lead to death, which is a huge threat to people’s life and property safety [55, 56]. Correlation analysis showed that AOD was proportional to NG. AOD in Xinjiang showed a downward trend, which was possibly related to the implementation of coal to NG conversion project. Although coal burning is no longer the dominant factor affecting air quality, other factors have also played a direct or indirect role. The development of Xinjiang has also caused pollution to the local environment. Therefore, we should not only rely on decisions by government but also establish the sense of protecting environment. Government needs to actively fund and study the new energy, change the energy structure, vigorously promote new energy, formulate laws and regulations to reduce industrial pollution emissions, rationally optimize the emission system of all kinds of vehicles, reduce tail gas emissions, advocate low-carbon travel, substantially abate the generation of pollution, and maximize the improvement of air quality. The general public should actively respond to the government’s calls on low-carbon travel, frequently participate in “planting and greening” and other activities, more importantly, establish their environmental awareness.

## Conclusion

The variation of AOD in Xinjiang is regional. The highest AOD value was found in southern Xinjiang. The AOD value in spring of southern Xinjiang (0.476) was significantly higher than that of northern Xinjiang (0.151) and eastern Xinjiang (0.156), and the AOD value in four seasons was significantly higher than that of other study regions. AOD in northern Xinjiang reached its highest value in winter (0.156). In addition, the maximum range of AOD in spring was more obvious than that in any other seasons.

The average value of AOD in Xinjiang was 0.196, showing a wavy trend. From 2000 to 2005, AOD showed a downward trend (0.206–0.158), and then increased significantly (0.228). After 2014, AOD showed a stable downward trend. Through RF model analysis, AOD in Xinjiang is affected by different natural and human factors, and the main result is the joint action of natural and human factors. NG is the most significant influencing factor (14.65%), followed by P (13.65%). Other influencing factors also affect AOD directly or indirectly.

HYSPLIT model is applied to imitate the forward and backward trajectory of the spring in the region with high AOD value (southern Xinjiang). Aerosol in southern Xinjiang is related to the short distance transport of dust aerosol from the Taklimakan Desert. These dust aerosols can affect the Inner Mongolia region of China through long distance transport. The Tianshan Mountains can block the transport and contribution of dust aerosols to northern Xinjiang.

## Supporting information

S1 File Raw data. It contains AOD values of the year and month.
(XLSX)
